# AI-enhanced liquid biopsy analysis for real-time monitoring of circulating tumor DNA mutations during targeted therapy resistance development

**DOI:** 10.1097/MS9.0000000000004155

**Published:** 2025-10-27

**Authors:** Semil Saleem, Muhammad Talha, Mahnoor Fatima, Fahad Khan, Abdullah Imtiaz

**Affiliations:** a Department of Medicine, King Edward Medical University, Lahore, Pakistan; b Department of Medicine, Shaheed Ziaur Rahman Medical College, Rajshahi Medical University, Bogura, Bangladesh; c Department of Medicine, Wah Medical College, Wah Cantt, Pakistan

*To the Editor,*

Targeted cancer therapies, administered to patients with malignancies, have revolutionized the entire therapeutic armamentarium of cancer and improved patient outcomes. Unfortunately, resistance to those therapies has become a most potent challenge, wherein resistance helps in tumor progression and limits long-term efficacy. Bias in tumor profiling via traditional tissue biopsy, which is intrusive, therefore, cannot keep up with the dynamic aspects of genetic evolution that are critical in the development of resistance. On the other hand, liquid biopsy with circulating tumor DNA (ctDNA) and its analysis is minimally invasive and clinically useful in real-time monitoring of intratumor heterogeneity and mutation profiles. Artificial intelligence (AI) and machine learning algorithms have also recently been put to work to complement ctDNA assays and significantly enhance their sensitivity, specificity, and prognostic usefulness toward a view wherein resistance mutations are swiftly distanced and treatment strategy acted upon by it well-timed in clinical oncology^[1,2]^. This is in line with the TITAN Guidelines on the need for transparency in AI use in healthcare^[3]^.

Liquid biopsy assays powered by AI involve the continuous tracking of variations at the level of tumor-derived ctDNA fragments using machine-learning algorithms. These machine-learning algorithms utilize highly endogenous multidimensional omics data that include genomic, transcriptomic, and proteomic information obtained, as they are extracted mostly from blood samples, in order to gain the ability to discern subtle mutation patterns as well as distinguish emerging resistance-driver alterations with previously unmatched precision. For instance, supervised learning models used in relation to ctDNA and proteomic data from patients with non-small-cell lung cancer indicated a hazard ratio of 4.5 for overall survival and 2.27 for progression-free survival (*P* < 0.0001) for predictions of immunotherapy response. Moreover, combining AI with next-generation sequencing technologies improved the discrimination of real low-frequency mutations from artifacts caused by sequencing, addressing the earlier obstacles against minimal residual disease and early signatures of resistance in a variety of malignancies. This permits an oncologist to foresee treatment failure and subsequently switch treatment regimens ahead of time, thereby likely extending progression-free intervals and overall survival^[2,4,5]^.

There is an emerging body of evidence corroborating a strong correlation between ctDNA mutation dynamics and clinical markers of progression in patients receiving targeted therapy. AI-enabled longitudinal monitoring of ctDNA profiles reveals heterogeneity and temporal changes in mutation landscapes that correlate with radiological and biochemical measures of tumor burden and therapeutic response. Molecules potentially associated with resistance, such as secondary mutations in *EGFR, KRAS*, or *BRAF* genes, have been detected weeks to months prior to clinical relapse and provide a narrow therapeutic window for intervention, sometimes only a few weeks. Such understandings allow for the formation of adaptive treatment algorithms such that modulation of therapy is based on ctDNA evolution, thus personalizing to the tumor biology of each patient. Custom-made therapy approaches toward this end follow the precision oncology paradigm, allowing for maximal therapeutic effect while avoiding unnecessary toxicity from treatments unlikely to work^[6,7]^.

The integration of AI-based liquid biopsy into the existing patterns of cancer care practices requires the collaboration of multiple disciplines to standardize the processes of testing and validate predictive models aimed at evidence-based expression of clinical utility across cancer types. ctDNA monitoring is becoming an endpoint more often in clinical trials designed to monitor resistance and assess the first response, which is an indication of a growing awareness of its role in precision medicine. By sharing ctDNA data and the insights generated by AI in real time through digital health platforms, oncology teams can be supported in making evidence-based treatment decisions. To completely recognize the promise of this technology, it is crucial that we provide education for oncology practitioners. This includes training on how to interpret the molecular data and to implement adaptive treatment algorithms into their practice^[6,8]^.

To conclude, the use of cognitive computing in the determination of ctDNA in liquid-phase biopsies stands out to be a milestone in continuously regulating the resistance to targeted therapy. Via this capability of determining the resistance mutations at early stages, giving rise to effective treatment protocols, this step seems to be a revolution in cancer care. Thus, sustained and committed efforts in clinical evaluation, protocol implementation, and providing guidance and instructions to practitioners will be fundamental in maximizing patient outcomes in personalized cancer treatment.

## Data Availability

Not applicable to this study.

## Research registration unique identifying number (UIN)

No human subjects were involved.
